# Genomic landscape and survival analysis of ctDNA “neo-*RAS* wild-type” patients with originally *RAS* mutant metastatic colorectal cancer

**DOI:** 10.3389/fonc.2023.1160673

**Published:** 2023-03-29

**Authors:** Chiara Nicolazzo, Valentina Magri, Luca Marino, Francesca Belardinilli, Federica Di Nicolantonio, Gianluigi De Renzi, Salvatore Caponnetto, Michela De Meo, Giuseppe Giannini, Daniele Santini, Enrico Cortesi, Paola Gazzaniga

**Affiliations:** ^1^ Lab. Liquid Biopsy, Department of Molecular Medicine, Sapienza University of Rome, Rome, Italy; ^2^ Department of Radiology, Oncology and Pathology, Sapienza University, Rome, Italy; ^3^ Department of Mechanical and Aerospace Engineering, Sapienza University of Rome, Rome, Italy; ^4^ Department of Oncology, University of Torino, Candiolo, Italy

**Keywords:** neo-*RAS*, *RAS* mutant colorectal cancer, survival, bevacizumab, circulating tumor DNA

## Abstract

**Background:**

The term “neo-*RAS* wild-type” refers to the switch to *RAS* wild-type disease in plasma circulating tumor DNA (ctDNA) from originally *RAS* mutant colorectal cancers. Consistently, the hypothesis to re-determine *RAS* mutational status in ctDNA at disease progression in RAS mutant mCRC opened to a new perspective for clinically-based selection of patients to be treated with EGFR inhibitors. Currently, the genomic landscape of “neo-*RAS* wild-type” is unknown. This is a prospective study aimed to investigate clinical and genomic features associated with *RAS* mutation clearance in a large cohort of *RAS* mutant mCRC patients who converted to *RAS* wild- type in liquid biopsy at failure of first-line treatments. Secondary aim was to investigate the long term prognostic significance of “true neo-*RAS wild*- type”.

**Patients and methods:**

70 patients with stage IV *RAS* mutant colorectal cancer were prospectively enrolled. Plasma samples were collected at progression from first-line treatment. *RAS*/BRAF mutations in plasma were assessed by RT-PCR. In *RAS*/BRAF wild-type samples, ctDNA was used to generate libraries using a 17 genes panel whose alteration has clinical relevance. To investigate the prognostic significance of *RAS* mutation clearance, test curves for PFS and OS were represented by Kaplan-Meier estimator plot and Log-rank test.

**Results:**

The most commonly detected actionable mutations in “neo-*RAS* wild-type” were: *PIK3CA* (35.7%); *RET* (11.9%); *IDH1* (9.5%); *KIT* (7%); *EGFR* (7%); *MET* (4.7%); *ERBB2* (4.7%); *FGFR3* (4.7%). Both OS and post-progression survival were longer in patients with “neo-*RAS* wild-type” compared to those who remained *RAS* mutant (p<0.001 for both).

**Conclusions:**

De-novo-targetable mutations occured in a large percentage of “neo-*RAS* wild-type”, being *PIK3CA* the most commonly detected. *RAS* mutation clearance in ctDNA is associated with long- term improvement of overall survival.

## 1 Introduction

Patients with K*RAS*/N*RAS* mutant metastatic colorectal cancer(mCRC) have poorer clinical outcome compared to those with *RAS* wild- type disease. Current treatment is based on combinations of chemotherapy and antiangiogenic agents with limited efficacy upon disease progression. Since the assessment of *RAS* mutational status currently guides the therapeutic choice, tissue biopsy in patients with mCRC is mandatory (1). Despite tissue biopsy still represents the gold standard, circulating tumor DNA (ctDNA) analysis in plasma is increasingly used to monitor disease status and assess treatment efficacy in real time, qualifying as a potential tool to re-define patient care (2). Although comprehensive genomic profiling in plasma has identified potential targets for therapeutic intervention in *RAS* wild- type mCRC, no targets for anticancer therapy have been confirmed in *RAS* mutant mCRC (3). In the last years, evidence has been provided that *RAS*-mutant clones are more likely to be cleared in plasma. This phenomenon, currently known as “neo-*RAS* wild-type (wt)”, leads to the appearance of a frame-time characterized by *RAS* wt disease in plasma, which has been described in course of treatment and at the time of disease progression, according to studies (4–6). This observation led to the hypothesis that *RAS* assessment in ctDNA at disease progression could change the clinically-based selection of patients to be treated with EGFR inhibitors (7–11). The genomic landscape of actionable alterations in “neo- *RAS* wt” patients has never been investigated to date. Further, studies recently aimed to investigate the phenomenon of “neo-*RAS* wt” suggest an association between clearance of *RAS* mutations in ctDNA and clinical outcome (12, 13). Nevertheless, these studies are flowed by short follow up period, as well as insufficient number of patients with events for survival analysis and lack of evidence of ctDNA presence in plasma samples. This is a prospective study aimed to investigate clinical and genomic features associated with *RAS* mutation clearance in a large cohort of *RAS* mutant mCRC patients who converted to *RAS* wt in liquid biopsy at failure of first-line treatments. Secondary aim was to investigate the long term prognostic significance of “true neo-*RAS* wt”.

## 2 Patients and methods

### 2.1 Patients and plasma samples preparation

**Figure 1:**
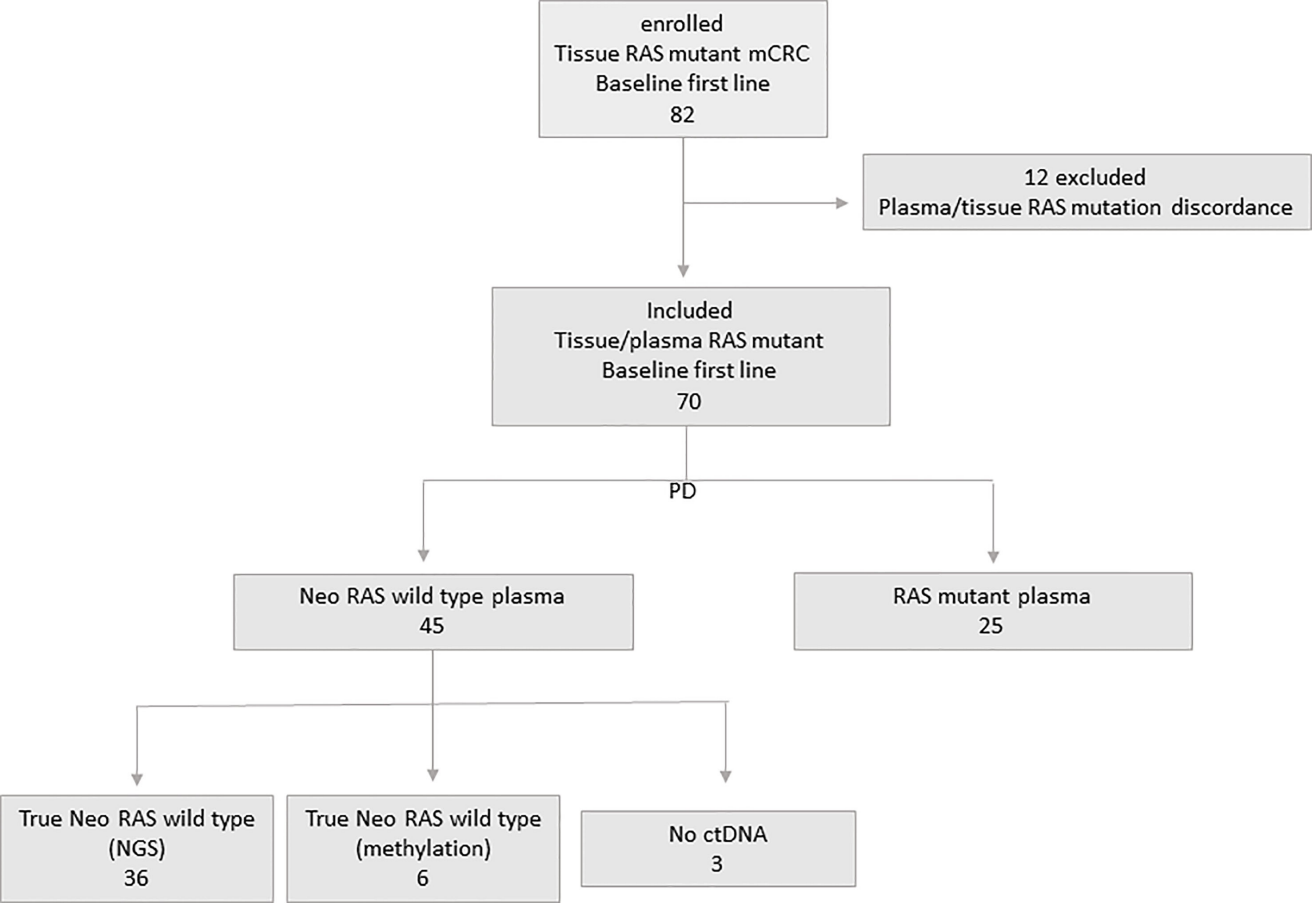
Flowchart of the study.

The study included 82 consecutive patients with unresectable *RAS* mutant stage IV colorectal cancer enrolled at Policlinico Umberto I between January 2017 and December 2021 with last follow-up on January 1st 2023. Overall survival (OS) was measured from diagnosis to the date of death, and surviving patients were censored on January 1st 2023. Progression free survival (PFS) was measured from the start of first-line treatment to progression disease event. Median follow up time was 50 months (range 26.7-66). Inclusion criteria were: males or females, age > 18 years; evidence of *RAS*/BRAF mutations as assessed in primary tumor tissue at the time of diagnosis; no previous lines of treatment received; ECOG performance status ≤2; signed informed consent. *RAS* mutational status at baseline was examined in formalin-fixed and parafinn-embedded tissue sections from primary tumors and in ctDNA according to standard procedures. Patients were treated with standard first-line chemotherapy with or without Bevacizumab according to national treatment guidelines. Clinical response was evaluated according to the Response Evaluation Criteria in Solid Tumours, V.1.1. Patients with discordant *RAS* mutational status in tissue and plasma samples were excluded. In the population of 70 enrolled patients, a further blood draw was performed at the time of disease progression. Blood draws were performed after obtaining informed consent. Authorization to perform liquid biopsies was released by the Regional Ethical Committee (No.:179/16) and the study was conducted in accordance with the Declaration of Helsinki. Plasma samples were obtained by centrifugation of 6 mL of blood at 1500 rpm for 10 min, followed by removal of plasma, which was further centrifuged at 13000 rpm for 1 min. Plasma samples were stored at -80°C until use. **Figure 1** shows the flowchart of the study.

### 2.2 Mutational analysis in plasma ctDNA

Idylla™(Biocartis NV, Mechelen, Belgium) was used to screen K*RAS*, N*RAS* and BRAF mutational status in plasma samples at progressive disease (PD). All *RAS*/BRAF wt samples were further analyzed through next generation sequencing (NGS) in order to confirm the presence of ctDNA. To this purpose, ctDNA was extracted from 1 ml of plasma for each patient through Maxwell RSC 96 Instrument (Promega corporation, Madison, WI, USA) employing Maxwell^®^ RSC ccfDNA Plasma Kit, according to the manufacturer’s instructions. Samples were quantified and their fragmentation index was assessed through EasyPGX qPCR instrument with the EasyPGX Analysis Software (Diatech Pharmacogenetics, Jesi, AN, Italy), according to the manufacturer’s protocol. Based on concentration and index fragmentation values, 25 µL of ctDNA were used to generate libraries using Myriapod NGS Cancer panel DNA kit (Diatech Pharmacogenetics) that allows the analysis of single nucleotide polymorphisms and indels in 17 genes (*ALK, BRAF, EGFR, ERBB2, FGFR3, HRAS, IDH1, IDH2, KIT, KRAS, MET, NRAS, PDGFRA, PIK3CA, POLE, RET, ROS1*) whose alteration has clinical relevance. Briefly, samples were amplified by multiplex-PCR using two mixtures of primers in order to obtain 101 fragments of length between 103 and 171 bases including the hot-spot regions of interest. After purification with magnetic beads to remove residual primers, an amplification indexing reaction was performed to bind to each fragment a unique pair of two bar codes specific for each sample and a specific adapter for the sequencing platform. Then, libraries were normalized by quantity using magnetic beads in order to ensure homogeneous coverage of the samples during the sequencing phase. Finally, the normalized libraries were pooled together and sequenced in parallel on the iSeq platform (Illumina Inc., San Diego, CA, USA), according to manufacturer’s instructions. Data analysis was performed using the Myriapod NGS Data Analysis Software (Diatech Pharmacogenetics). Samples with no other mutations detected through NGS were analysed through methylation assay to confirm the presence of ctDNA as previously described (4).

### 2.3 Statistical analysis

Categorical variables were reported as frequency distribution, whereas continuous variables were described through median and interquartile range. A Mann-Whitney U test was adopted to compare the measured values between the continuous variables. χ2 statistics and exact Fisher test were used to assess differences between categorical variables. Test curves for PFS and OS were represented by Kaplan-Meier estimator plot and a Log-rank test was performed to compare survival curves. A p-value less than 0.05 was considered statistically significant. All statistical tests were 2-sided. Statistical analysis was carried out by using IBM SPSS Statistics software (ver. 25.0).

## 3 Results

Twelve out of 82 patients with discordant tissue/plasma *RAS* mutational status at baseline were excluded. Seventy patients with concordant tissue/plasma *RAS* mutant primary mCRC at baseline were enrolled, with a median age of 62 years (47-88). All had stage IV disease at diagnosis (hepatic 90%). According to sidedness, we had 67% left-sided and 33% right-sided tumors. Characteristics of patient population are illustrated in **Table 1**.

**Table 1:** Patients population characteristics.

Age, years	
Mean	62
Range	47-88
Sex	n.(%)
Male	46 (66%)
Female	24 (34%)
Line of therapy	
1st	70 (100%)
Treatment received CT plus Bev CT alone	56 (80%) 14 (20%)
Location of primary tumor	
Left	47 (67%)
Right	23 (33%)
Histology	
Adenocarcinoma	70 (100%)
Metastatic sites	
liver	63 (90%)
other	7 (10%)
*RAS*/BRAFmutation tissue/plasma baseline	
Mutated K*RAS* G12D K*RAS* G12V K*RAS* G12A K*RAS* G12R/S K*RAS* G12C K*RAS* Q61 K*RAS* A146T K*RAS* G13D N*RAS* G12D N*RAS* G12C N*RAS* Q61R N*RAS* A146T BRAF V600E	70(100%) 16 (22%) 15 (21%) 5 (7%) 2 (3%) 6 (8%) 5 (7%) 5 (7%) 7 (10%) 3 (4%) 1(1%) 1 (1%) 1(1%) 3 (4%)

### 3.1 Assessment of *RAS* mutational status in plasma at PD

Liquid biopsy was performed at the time of disease progression from first line systemic treatment consisting in chemotherapy (CT) plus (80%) or without (20%) Bevacizumab. ctDNA analysis was performed to evaluate the rate of disappearance of *RAS* mutations at PD. According to plasma ctDNA analysis at PD, patients were initially defined as follows: 1) “ctDNA neo-*RAS* wt” (45/70, 64%); and 2)”ctDNA *RAS* mutant”(25/70, 36%). The presence of ctDNA in “neo-*RAS* wt” samples was confirmed through NGS analysis in 36/45 (80%) patients, found positive for at least one somatic mutation. Nine out of 45 “neo-*RAS* wt”samples (20%) had no other somatic mutations detected, and were therefore analyzed through methylation assay in order to confirm or exclude the presence of ctDNA. Six out of these 9 samples (67%) were found positive for methylation test, while 3/9 (33%) were found negative. These last were classified as “no ctDNA samples”. Therefore, through NGS or methylation assay we could confirm the presence of ctDNA in 42/45 (93%) “ctDNA neo-*RAS* wt” samples; these samples were defined as “ctDNA true neo-*RAS* wt”. In the group of Bevacizumab treated patients the rate of” neo-*RAS* wt” was 75% compared to 21% in the group of patients treated with CT alone. Bevacizumab was found significantly associated with *RAS* mutation conversion (χ2 test p<0.001). Doublet vs triplet CT was not associated with *RAS* mutation conversion (χ2 test p=0.69). Metastatic sites (hepatic vs non-hepatic) were found not associated to *RAS* mutation conversion (χ2 test p=0.67).

### 3.2 Targetable alterations in ctDNA from “true neo-*RAS* wild-type” patients compared to primary tumor tissues

**Figure 2:**
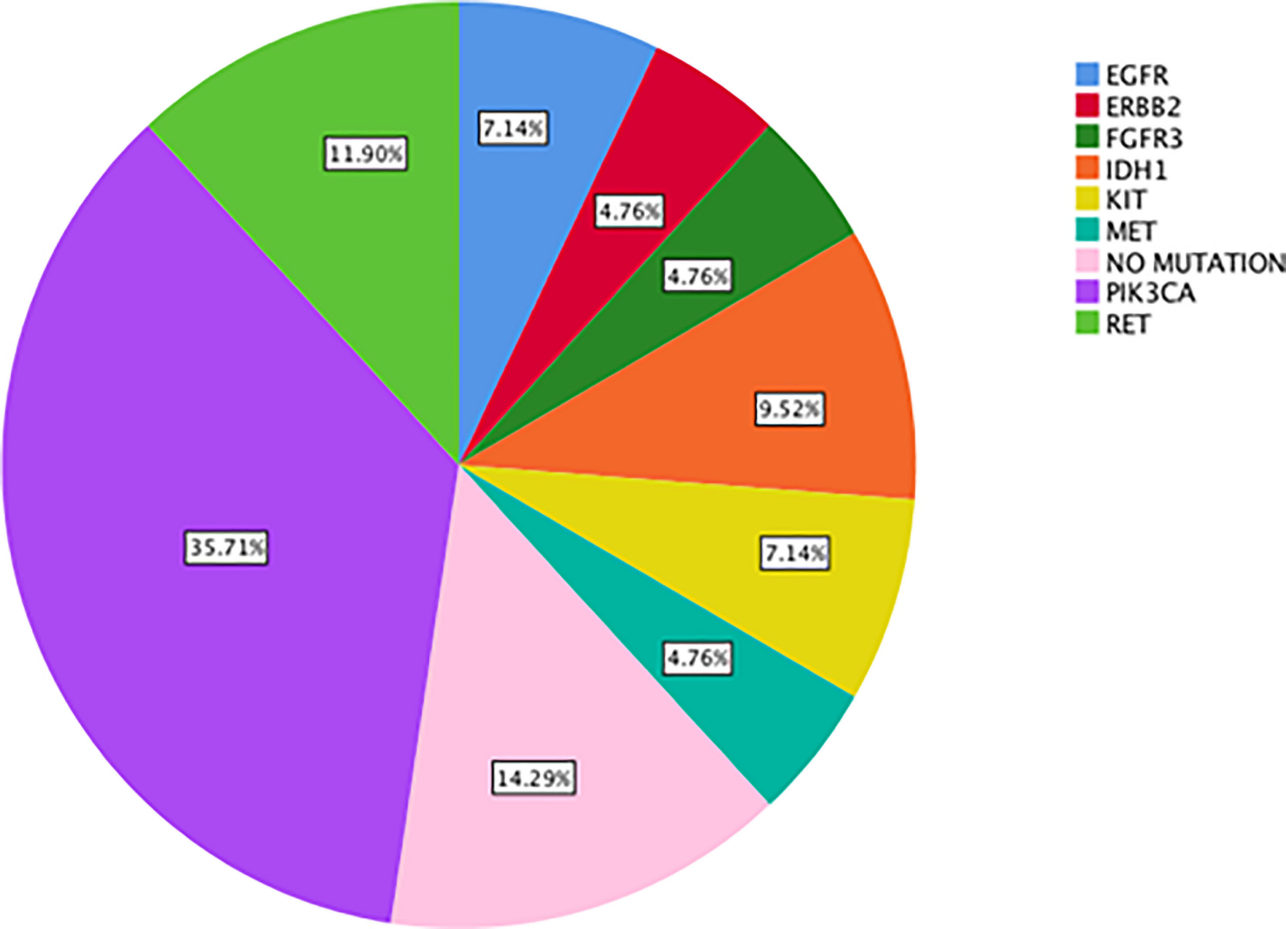
Percentage of actionable alterations in “true neo-*RAS* wild-type” ctDNA samples at progression of disease.

**Figure 3:**
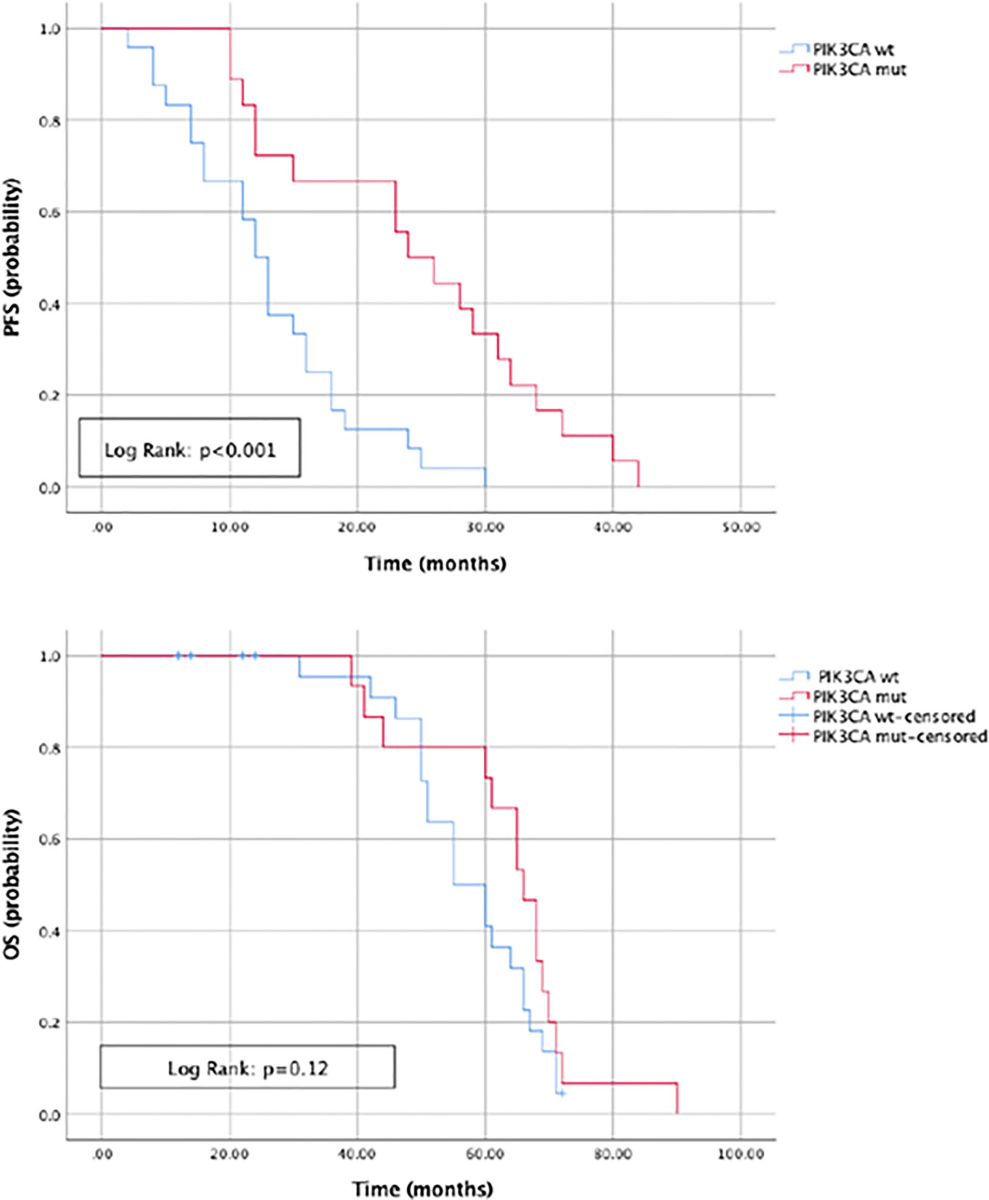
Progression free survival and Overall survival in patients with and without *PIK3CA* mutation in plasma ctDNA at progression of disease.

The most commonly detected somatic mutations in “true neo-*RAS* wt” at PD were: *PIK3CA* (35.7%); *RET* (11.9%); *IDH1* (9.5%); *KI*T (7%); *EGFR* (7%); *MET* (4.7%); *ERBB2* (4.7%); *FGFR3* (4.7%) (**Figure 2**). Among those, only *PIK3CA* and *FGFR3* mutations were already detectable in primary tumor tissues in 2/14 and in 2/2 samples respectively. All the other mutations detected in “neo-*RAS* wt” samples were not present in primary tumor tissue. Of note, in 14.3% of samples we did not detect any mutation. Nevertheless, the presence of methylated cancer specific sequences was suggestive for ctDNA presence. Since *PIK3CA* mutations were detected in 35% of “neo-*RAS* wt” patients, we could evaluate the association between *PIK3CA* mutations and clinical outcomes in “true neo-*RAS* wt. Median PFS was 24 months (17.7 -30.2) in patients with *PIK3CA* mutation in plasma ctDNA at PD versus 12 months (10.1 – 13.9) in patients with no evidence of *PIK3CA* mutation (p<0.001). OS in patients with and without *PIK3CA* mutation in plasma ctDNA at PD were 65 months (58.3 – 71.7) and 55 months (44.1 – 64.8) respectively (p=0.12) (**Figure 3**). The small number of patients with other mutations did not allow drawing any statistically significant conclusions.

### 3.3 Prognostic significance of *RAS* mutation conversion

**Figure 4:**
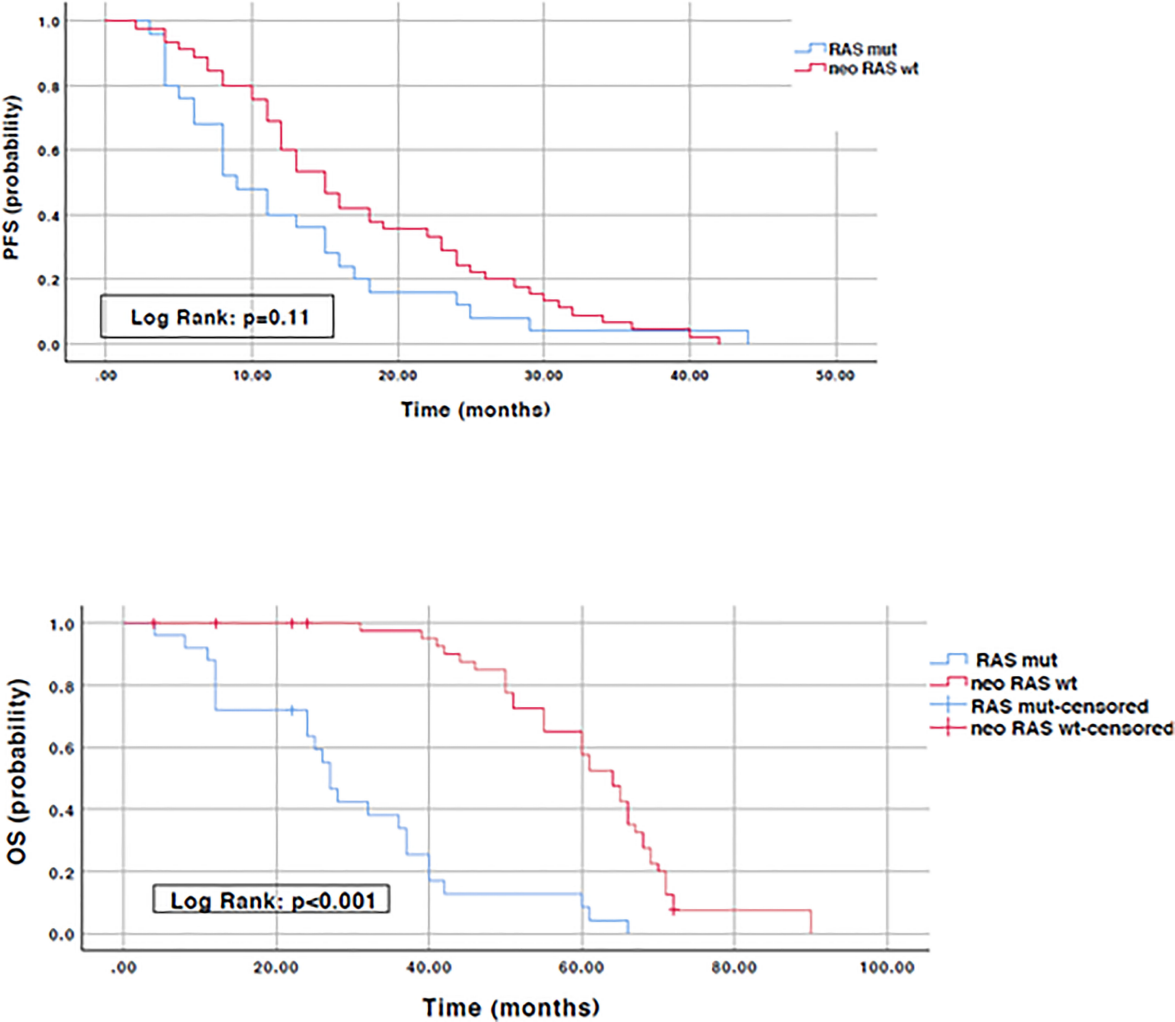
Progression free survival and Overall survival in “true neo-*RAS* wild-type” compared to *RAS* mutant.

**Figure 5:**
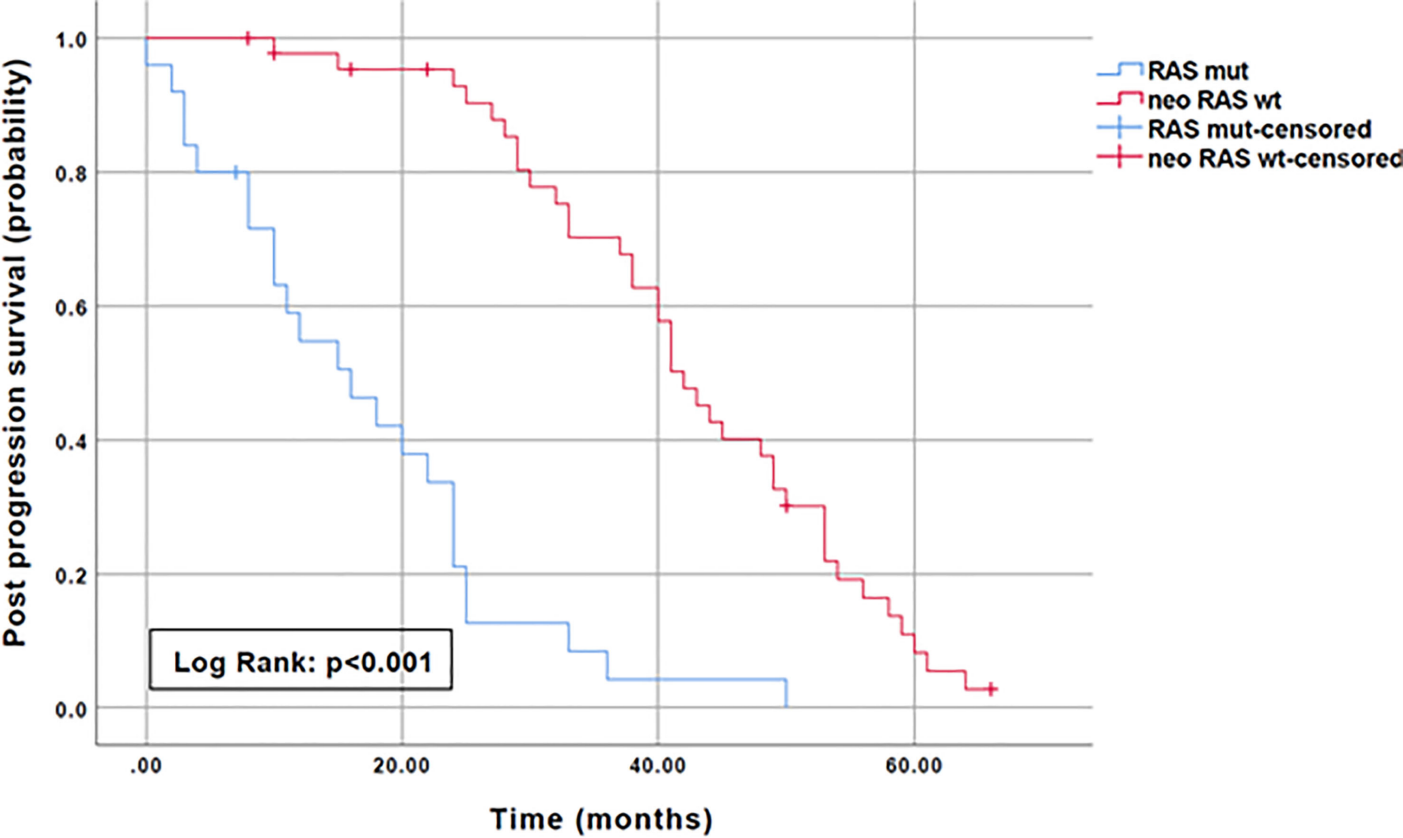
Post progression Overall Survival in “true neo-*RAS* wild-type” compared to *RAS* mutant.

To evaluate the prognostic impact of *RAS* mutation clearance, we analyzed the association between the dynamic changes in *RAS* status and clinical outcomes. The median PFS of patients with “true neo-*RAS* wt” was 15 months (11.7-18.3) compared to 9 months (5.2-12.5) of patients who maintained plasma *RAS* mutation at PD. This difference was not statistically significant (p=0.11). Median OS was 64 months in patients with “true neo-*RAS* wt” versus 27 months in patients who remained *RAS* mutant (p<0.001) (**Figure 4**). Median OS after first-line progression in “neo-*RAS* wt” patients was 42 months (37.8-46.2) compared to 16 months (7.7-24.3) of those who remained *RAS* mutant (p<0.001) (**Figure 5**).

### 3.4 Prognostic significance of *RAS* mutation conversion according to sidedness

There was no significant difference in the PFS and OS between left-sided tumors and right-sided tumors (median PFS: 13 (9.7 – 16.4) versus 13 (9.2 - 16.7) months, p=0.36; median OS: 55(43.8 – 66.8) versus 51(35.1 – 66.9) months, p=0.26). PFS and OS were then stratified by *RAS* mutation conversion and sidedness. In left-sided tumors median PFS was 13 months (8.8-17.2) in patients with “true neo-*RAS* wt” versus 11 months (4.8 -17.2) in patients who remained *RAS* mutant (p=0.36). OS was 61 months (54.2 – 67.8) in patients with “true neo-*RAS* wt” versus 32 months (19.4 – 44.6) in patients who remained *RAS* mutant (p<0.001). In right-sided tumors median PFS was 18 (12.2-23.7) in patients with “true neo-*RAS* wt” versus 8 months (5.7-10.3) in patients who remained *RAS* mutant (p=0.96). OS was 65 months (55.3 – 74.6) in patients with “true neo-*RAS* wt” versus 24 months (10.1 – 37.9) in patients who remained *RAS* mutant (p<0.001). In the group of “neo-*RAS* wt” median PFS in left- and right-sided tumors was not found statistically significant (p=0.48). In the group of “neo-*RAS* wt” median OS in left- and right-sided tumors was found not statistically significant (p=0.31) (data not shown).

## 4 Discussion

Since its first description (14), the concept of “neo-*RAS* wt” in colorectal cancer has gained increasing attention, providing new insights for following targeted treatment selection. In fact, the absence of any clinically relevant mutation of *RAS* genes in blood has been recently used as a therapeutically exploitable window to change the clinically-based selection of patients to be treated with EGFR inhibitors (7–11). The rate of *RAS* mutation clearance in plasma is highly variable, ranging from 8% to 70% of cases according to studies (15). The inconsistency of results among studies might be attributed to a misinterpretation of the term “*RAS* mutation clearance”. In fact, many studies are flowed by the lack of demonstration of ctDNA presence in “neo-*RAS* wt” plasma samples, frequently leading to confusion between “non-shedder” patients and those with true *RAS* mutation clearance. The finding of *RAS* wt in plasma without a proper confirmation of ctDNA presence in the sample might explain why most studies have reported that *RAS* mutation clearance in the ctDNA occurs early after treatment and is associated to better outcomes (12). *RAS* mutation disappearance at the time of disease progression has been poorly investigated to date. Sunakawa et al. recently reported that “neo-*RAS* wt” ctDNA appears early in course treatment and is associated with better outcome (13). Interestingly, authors report that *RAS* mutations remained undetectable during progression in 62% of patients. Nevertheless, the lack of confirmation of ctDNA presence makes it impossible to establish whether the longer survival was linked to a scarce release of ctDNA rather than a “true neo-*RAS* wt” disease. Furthermore, the short follow up period and the insufficient number of patients with events for survival analysis strongly affected the prognostic significance of “neo-*RAS* wt” ctDNA. Similarly, Wang et al. reported a relatively high rate of *RAS* clearance (42.6%) in mCRC patients before PD and reported better outcomes in patients with plasma *RAS*/BRAF clearance compared to those who remained *RAS*/BRAF mutant; nevertheless, the follow up time was not sufficient to confirm the prognostic impact of *RAS* clearance on OS (16). As far as we know, this is the largest cohort of “true *RAS* wt converters”, in which the follow up period was sufficiently long for a statistically significant correlation with OS. A further strenght of the study is that presence of ctDNA was consistently confirmed through NGS or methylation assays. Our choice to use a gene panel for detection of only clinically actionable mutations in ctDNA (thus lacking p53, frequently mutated in mCRC) might explain why in 9 cases it was necessary to use a methylation test to confirm the presence of ctDNA. We here report that “neo-*RAS* wt” occurs mainly in Bevacizumab treated patients, confirming our previous report that Bevacizumab in first-line is as an independent predictor of *RAS* mutation clearance in ctDNA at PD (17). Nevertheless, differently from that reported by Sunakawa et al, we disagree in that *RAS*-mutant colon cancer cells may have changed by intensive treatment, since in our cohort triplet vs doublet was not found associated with *RAS* mutation conversion. The biological rationale why Bevacizumab is associated to *RAS* mutation clearance is currently unknown. This is the first study describing the molecular landscape of actionable alterations in “true neo-*RAS* wt” ctDNA samples at progression of disease. Compared with baseline tissue samples, ctDNA samples from “neo-*RAS* wt” patients displayed alterations not present at the diagnostic biopsy. Specifically, only FGFR3 mutation had been already detected in primary tumor tissues, while *PIK3CA* was acquired in 28% of cases. Of note, *PIK3CA* mutation was associated with prolonged PFS but not with OS in “neo-*RAS* wt” patients. Although the prognostic significance of *PIK3CA* mutations in mCRC is still controversial, evidence has been provided concerning a positive impact of *PIK3CA* mutation on patients outcome (18–20). This observation might be related to an increased proportion of microsatellite instability (MSI)-high in patients with *PIK3CA* mutations. Unfortunately we lack information concerning MSI status in our patients cohort. Although the identification of gain-of-function *RET* mutations have been described in colorectal cancer (21), the clinical significance of *RET* mutations detected in our cohort is currently unknown, with the exception of variant G691S, detected in 3 cases, which has been reported associated to increased invasion in cultured cells (22). Activating point mutations in *c-KIT* are well-documented in gastrointestinal stromal tumors and in colorectal cancer tissues, where the mutation positive rate was found 19% according to a recent report (23). Interestingly, we detected *IDH*1/2 mutations in 7% of “neo-*RAS* wt” plasma samples. Although the incidence of *IDH*1/2 mutations in colorectal cancers is <1%, evidence supports *IDH*1/2 mutations as drivers during the early evolutionary phase of tumorigenesis (24). Evidence has been provided that *IDH*1/2 mutants are more frequent in colorectal cancers showing CpG island methylator phenotype, often concurrent with K*RAS*/*BRAF* mutations, although with a lower allelic frequency (25). The detection of *IDH*1 mutations in 7% of “neo-*RAS* wt” patients at PD might be suggestive for a clonal selection of *IDH*1 mutant subclones through the course of the disease. Nevertheless, since we did not perform parallel analysis of mononuclear blood cells we cannot exclude that these new mutations not present in tumor biopsy could be ascribed to clonal hematopoiesis. Somatic mutation *EGFR* c.2062-3delC was detected in 7% of “neo-*RAS* wt”, although no functional evidence was reported in ClinVar for this variation. *MET*, *ERBB2* and *FGFR3* were detected in a minority of “neo-*RAS* wt” samples. This study has the following limitations. First, a parallel sequencing of mononuclear cells isolated from peripheral blood would have been important to exclude clonal hematopoiesis at least for rare mutations detected in ctDNA but not in tumor biopsy. Second, we did not measure the impact of second-line treatments on overall survival. Third, we did not evaluate the clinical impact of *EGFR* inhibitors in “neo-*RAS* wt” patients. We provided evidence that in originally *RAS* mutant mCRC the loss of *RAS* mutation at failure of first-line treatments strongly impacts on OS, suggesting that the clearance of *RAS* mutation at disease progression may serve as a reliable prognostic tool. The clearance of *RAS* mutations in blood was found associated with Bevacizumab use, independent of the intensity of CT (doublet vs. triplet). We further deepened for the first time the genomic landscape of “neo-*RAS* wt” patients and identified some actionable targets, although the clinical significance of these alterations need to be further evaluated in a larger sample size. If we assume that liquid biopsy performed at disease progression allows to reclassify an originally RAS mutant mCRC as a “neo RAS wild type”, our study might have several clinical implications.The most obvious is the hypothesis that “neo RAS wild type” patients might benefit from EGFR blockade; this speculation is currently under investigation in ongoing clinical trials (ClinicalTrials.gov Identifier: NCT04554836; NCT04189055). Furthermore,the identification of potential targetable alteration in many “neo *RAS* wt”patients might provide implications for subsequent treatments as illustrated in **Table 2**. For example, the high frequency of patients with acquired *PIK3CA* mutation in the” neo *RAS* wt” window might open up new perspectives for combined treatments with MEN1611, a *PI3KCA* Inhibitor, and cetuximab, currently under investigation in PRECISE-01 phase Ib/II study in patients with *RAS* wt mCRC (26). Further, *HER-2* is an emerging biomarker in colorectal cancer with the continuous evolution of specific anti-*HER2* therapeutic agents. Many clinical trials with different *HER2*-targeted agents are ongoing and have shown promising results in mCRC to date (27). Evidence has been provided that *HER2* mutations cause constitutional activation of proliferation signals, similarly to *HER2* gene amplification. Since in our population, 4.7% of neo *RAS* wt patients acquired *HER*-2 mutations, the hypothesis that these patients might benefit from *HER-2* targeted agents, although speculative, deserves further attention. In addition, although *RET* alterations are uncommon in mCRC it has been suggested that patients with *RET* alterations might potentially achieve benefit from *RET* targeting strategies (28). Surprisingly, in our series,we reported acquired *RET* mutations in 11% of “neo *RAS* wt”.*RET* point mutations have been described in different cancers, such as breast cancer,colorectal adenocarcinoma and gastrointestinal stromal tumors. Whether *RET* mutant mCRC might benefit from *RET* targeting drugs in mCRC is currently unknown, although *in vitro* studies have demonstrated that the *RET* inhibitor vandetanib abolishes the effects exerted by *RET*-533C mutant in transfected HEK293 cells (20). Furthermore, despite *IDH*1/2 mutations detected in the “neo *RAS* wt” patients have been recently reported as potential trunk drivers suitable for targeted therapy in CRC (23), further studies are needed to understand their potential clinical significance. To date, *IDH*1 and *IDH*2 inhibitors have been approved in acute myeloid leukemia and more recently in cholangiocarcinoma; nevertheless some clinical trials for other solid tumors with evidence of *IDH*1/2 mutations, including mCRC, are ongoing. Similarly, two phase II trials are currently investigating the effect of avapritinib and pemigatinib in solid malignancies with *cKIT* and *FGFR* mutations respectively.Finally,a phase Ib, multicenter clinical trial is ongoing to determine the efficacy of the *c-MET* inhibitor INC280 in combination with cetuximab. In conclusion, this study might represent the basis for future clinical trials aimed to paradigm-changing clinical applications of ctDNA in *RAS* mutant colorectal tumors in a perspective of precision oncology.

**Table 2:** Ongoing clinical trials hypothetically suitable in the “neo *RAS* wt” patients according to the specific mutations detected in ctDNA.

Molecular Alteration	On going Clinical Trials
	NCT04495621:MEN16AA With Cetuximab in Metastatic Colorectal Cancer (C-PRECISE-01)
HER-2	NCT03457896: Study of Netatinib + Trastuzumab or Neratinib + Cetuximab in Patients With KRAS/NRAS/BRAS/PIK3CA Wild-Type Metastatic Colorectal Cancer by HER2 Status
RET	NCT03037385: Phase1/2 Study of the Highly- selective RET Inhinitor, Pralsetinib (BLU-667), in Particpants With Thyroid Cancer, Non-Small Cell Lung Cancer, and Other Advanced Solid Tumors (ARROW)
IDH1/2	NCT04584008: Targeted Agent Evaluation in Digestive Cancerns in China Based on Molecular Characteristics (VISIONARY)
KIT	NCT04771520: Avapritnib for thr Treatment of Metastatic or Unresectable Colorectal Cancer Harboring FGFR Alterations
MET	NCT02205398: Study of Safety and Efficacy of INC280 and Cetuximab, in Adult c-MET Positive mcRC and HNSCC Patients After Progression on Cetuximab or Panitumumab Therapy

## Data availability statement

The datasets presented in this article are not readily available for privacy issue. Requests to access the datasets should be directed to corresponding author.

## Ethics statement

The studies involving human participants were reviewed and approved by Regional Ethical Committee, Policlinico Umberto I (No.:179/16). The patients/participants provided their written informed consent to participate in this study.

## Author contributions

PG and EC performed study concept and design. VM and SC performed acquisition of clinical data. GD and MD performed ctDNA extraction from blood samples.VM and CN performed RT-PCR and NGS assays in plasma. FB and GG performed NGS analysis in tissue samples. FD performed methylation assay in plasma samples. VM, CN, EC and PG performed interpretation of data. LM performed statistical analysis. PG, EC and DS performed drafting of the manuscript. PG and EC supervised the study. PG and DS wrote the manuscript. All authors read and approved the final manuscript. All authors contributed to the article and approved the submitted version.

## Memoriam

“I don't think anyone dies. I believe that the soul actually becomes a shadow and at the height of its wandering lies down at the foot of an unseen flower. Those yellow flowers the countryside is full of, whenyou come home and you wish she existed.” This paper is dedicated to the memory of Gianfranco Spalletta.

## References

[1] Ciardiello F Ciardiello D Martini G Napolitano S Tabernero J Cervantes A. Clinical management of metastatic colorectal cancer in the era of precision medicine. CA: Cancer J Clin (2022) 72(4):372–401. doi: 10.3322/caac.21728 35472088

[2] Zhou H Zhu L Song J Wang G Li P Li W. Liquid biopsy at the frontier of detection, prognosis and progression monitoring in colorectal cancer. Mol Cancer (2022) 21(1):86. doi: 10.1186/s12943-022-01556-2 35337361 PMC8951719

[3] Kato S Schwaederlé MC Fanta PT Okamura R Leichman L Lippman SM. Genomic assessment of blood-derived circulating tumor DNA in patients with colorectal cancers: correlation with tissue sequencing, therapeutic response, and survival. JCO Precis Oncol (2019) 3:1–16. doi: 10.1200/PO.18.00158 PMC6484865 31032472

[4] Nicolazzo C Barault L Caponnetto S De Renzi G Belardinilli F Bottillo I. True conversions from RAS mutant to RAS wild-type in circulating tumor DNA from metastatic colorectal cancer patients as assessed by methylation and mutational signature. Cancer Lett (2021) 507:89–96. doi: 10.1016/j.canlet.2021.03.014 33744389

[5] Klein-Scory S Wahner I Maslova M Al-Sewaidi Y Pohl M Mika T. Evolution of RAS mutational status in liquid biopsies during first-line chemotherapy for metastatic colorectal cancer. Front Oncol (2020) 10:1115. doi: 10.3389/fonc.2020.01115 32766143 PMC7378792

[6] Moati E Blons H Taly V Garlan F Wang-Renault SF Pietrasz D. Plasma clearance of RAS mutation under therapeutic pressure is a rare event in metastatic colorectal cancer. Int J Cancer (2020) 147(4):pp.1185–1189. doi: 10.1002/ijc.32657 31472013

[7] Raimondi C Nicolazzo C Belardinilli F Loreni F Gradilone A Mahdavian Y. Transient disappearance of RAS mutant clones in plasma: a counterintuitive clinical use of EGFR inhibitors in RAS mutant metastatic colorectal cancer. Cancers (2019) 11(1):p.42. doi: 10.3390/cancers11010042 PMC6357143 30621206

[8] Nicolazzo C Belardinilli F Caponnetto S Gradilone A Cortesi E Giannini G. Why the therapeutic impact of RAS mutation clearance in plasma ctDNA deserves to be further explored in metastatic colorectal cancer. Front Oncol (2019) 9:1414. doi: 10.3389/fonc.2019.01414 31921671 PMC6933952

[9] Gazzaniga P Nicolazzo C Caponnetto S Cortesi E. About RAS mutation clearance in plasma ctDNA from RAS-mutant colorectal cancer patients. JCO Precis Oncol (2021) 5:389–90. doi: 10.1200/PO.20.00376 34994600

[10] Osumi H Ishizuka N Takashima A Kumekawa Y Nakano D Shiozawa M. Multicentre single-arm phase II trial evaluating the safety and effiCacy of panitumumab and iRinOtecan in NeoRAS wild-type mEtaStatic colorectal cancer patientS (C-PROWESS trial): study protocol. BMJ Open (2022) 12(9):e063071. doi: 10.1136/bmjopen-2022-063071 PMC9438189 36581973

[11] Montes AF Lago NM de la Cámara Gómez J Rúa MC Castiñeiras AC Villarroel PG. Folfiri plus panitumumab as second-line treatment in mutated ras metastatic colorectal cancer patients who converted to wild type ras after receiving first-line FOLFOX/CAPOX plus bevacizumab-based treatment: phase II CONVERTIX trial. Ann Oncol (2019) 30:iv23–4. doi: 10.1093/annonc/mdz155.088

[12] Bouchahda M Saffroy R Karaboué A Hamelin J Innominato P Saliba F. Undetectable RAS-mutant clones in plasma: Possible implication for anti-EGFR therapy and prognosis in patients with RAS-mutant metastatic colorectal cancer. JCO Precis Oncol (2020) 4:1070–9. doi: 10.1200/PO.19.00400 PMC7529530 33015528

[13] Sunakawa Y Satake H Usher J Jaimes Y Miyamoto Y Nakamura M. Dynamic changes in RAS gene status in circulating tumour DNA: a phase II trial of first-line FOLFOXIRI plus bevacizumab for RAS-mutant metastatic colorectal cancer (JACCRO CC-11). ESMO Open (2022) 7(3):100512. doi: 10.1016/j.esmoop.2022.100512 35688061 PMC9271512

[14] Gazzaniga P Raimondi C Urbano F Cortesi E. Second line EGFR-inhibitors in RAS mutant metastatic colorectal cancer: The plasma RAS wild type “window of opportunity”. Ann Oncol (2018) 29:viii183–viii184. doi: 10.1093/annonc/mdy281.095

[15] Osumi H Vecchione L Keilholz U Vollbrecht C Alig AH Jobst C. NeoRAS wild-type in metastatic colorectal cancer: Myth or truth?–case series and review of the literature. Eur J Cancer (2021) 153:86–95. doi: 10.1016/j.ejca.2021.05.010 34153718

[16] Wang F Huang YS Wu HX Wang ZX Jin Y Yao YC. Genomic temporal heterogeneity of circulating tumour DNA in unresectable metastatic colorectal cancer under first-line treatment. Gut (2022) 71(7):1340–9. doi: 10.1136/gutjnl-2021-324852 PMC9185813 34489309

[17] Nicolazzo C Belardinilli F Vestri A Magri V De Renzi G De Meo M. RAS mutation conversion in bevacizumab-treated metastatic colorectal cancer patients: A liquid biopsy based study. Cancers (2022) 14(3):802. doi: 10.3390/cancers14030802 35159069 PMC8833999

[18] Mei ZB Duan CY Li CB Cui L Ogino S. Prognostic role of tumor PIK3CA mutation in colorectal cancer: a systematic review and meta-analysis. Ann Oncol (2016) 27(10):1836–48. doi: 10.1093/annonc/mdw264 PMC5035784 27436848

[19] Lin EI Tseng LH Gocke CD Reil S Le DT Azad NS. Mutational profiling of colorectal cancers with microsatellite instability. Oncotarget (2015) 6(39):p.42334. doi: 10.18632/oncotarget.5997 PMC4747229 26517354

[20] Han J Shi J Jiang M Zhang R Jin S Jia Y. Clinical characteristics and prognostic values of PIK3CA mutation in .Colorectal cancer patients. J Cancer Sci Clin Ther (2022) 6(1):121–32.

[21] Mendes Oliveira D Grillone K Mignogna C De Falco V Laudanna C Biamonte F. Next-generation sequencing analysis of receptor-type tyrosine kinase genes in surgically resected colon cancer: identification of gain-of-function mutations in the RET proto-oncogene. J Exp Clin Cancer Res (2018) 37(1):1–12. doi: 10.1186/s13046-018-0776-5 29665843 PMC5905113

[22] Sawai H Okada Y Kazanjian K Kim J Hasan S Hines OJ. The G691S RET polymorphism increases glial cell line–derived neurotrophic factor–induced pancreatic cancer cell invasion by amplifying mitogen-activated protein kinase signaling. Cancer Res (2005) 65(24):11536–44. doi: 10.1158/0008-5472.CAN-05-2843 16357163

[23] Ye J Lin M Zhang C Zhu X Li S Liu H. Tissue gene mutation profiles in patients with colorectal cancer and their clinical implications. Biomed Rep (2020) 13(1):43–8. doi: 10.3892/br.2020.1303 PMC7238404 32440349

[24] Huang J Tseng LH Parini V Lokhandwala PM Pallavajjala A Rodriguez E. IDH1 and IDH2 mutations in colorectal cancers. Am J Clin Pathol (2021) 156(5):777–86. doi: 10.1093/ajcp/aqab023 33929516

[25] Whitehall VLJ Dumenil TD McKeone DM Bond CE Bettington ML Buttenshaw RL. Isocitrate dehydrogenase 1 R132C mutation occurs exclusively in microsatellite stable colorectal cancers with the CpG island methylator phenotype. Epigenetics (2014) 9(11):.1454–1460. doi: 10.4161/15592294.2014.971624 PMC4622530 25496513

[26] Tabernero J Elez Fernandez E Ghiringhelli F Folprecht G Curigliano G Siena S. PRECISE-01 study: A phase Ib/II trial of MEN1611, a PI3K inhibitor, and cetuximab in patients with PIK3CA mutated metastatic colorectal cancer failing irinotecan, oxaliplatin, 5-FU and anti-EGFR containing regimens. Ann Oncol (2020) 31(3):S115. doi: 10.1016/j.annonc.2020.04.161

[27] Torres-Jiménez J Esteban-Villarrubia J Ferreiro-Monteagudo R. Precision medicine in metastatic colorectal cancer: Targeting ERBB2 (HER-2) oncogene. Cancers (Basel) (2022) 14(15):3718. doi: 10.3390/cancers14153718 35954382 PMC9367374

[28] Pietrantonio F Di Nicolantonio F Schrock AB Lee J Morano F Fucà G. RET fusions in a small subset of advanced colorectal cancers at risk of being neglected. Ann Oncol (2018) 29(6):1394–401. doi: 10.1093/annonc/mdy090 29538669

